# Green Synthesis, Characterization and Pharmaceutical Applications of Biocompatible Zinc Oxide Nanoparticles Using *Heliotropium rariflorum* Stocks

**DOI:** 10.3390/ph17111457

**Published:** 2024-10-31

**Authors:** Noor Ul Uza, Ghulam Dastagir, Syed Tanveer Shah, Elitsa Pavlova, Aftab Jamal, Mahmoud F. Seleiman, Jakub Černý

**Affiliations:** 1 Department of Botany, University of Peshawar, Peshawar 25130, Khyber Pakthunkhwa, Pakistan; noorbotany123@gmail.com (N.U.U.); dastagirbotany@yahoo.com (G.D.); 2 Department of Agriculture, Faculty of Biological and Health Sciences, Hazara University, Mansehra 21300, Khyber Pakthunkhwa, Pakistan; dr.syedtanveershah@hu.edu.pk; 3 Optics and Spectroscopy Department, Faculty of Physics, Sofia University St. Kliment Ohridski, 5 James Bourchier Blvd., 1164 Sofia, Bulgaria; elli_pavlova@abv.bg; 4 Key Laboratory of Arable Land Conservation (Middle and Lower Reaches of the Yangtze River), Ministry of Agriculture, College of Resources and Environment, Huazhong Agricultural University, Wuhan 430070, China; 5 Department of Plant Production, College of Food and Agriculture Sciences, King Saud University, P.O. Box 2460, Riyadh 11451, Saudi Arabia; mseleiman@ksu.edu.sa; 6 Department of Silviculture, Forestry and Game Management Research Institute, Na Olivě 550, 517 73 Opočno, Czech Republic

**Keywords:** antibacterial, antifungal, antioxidant, acute toxicity, analgesic, antipyretic, sedative

## Abstract

**Background:** Zinc oxide nanoparticles are safe, non-toxic, and biocompatible. These NPs are used in food packaging materials, self-cleaning glass, ceramics, deodorants, sunscreens, paints, coatings, ointments, lotions, and as preservatives. This study explored the biological potential of ZnO nanoparticles synthesized using *H. rariflorum*. **Methods:** In vitro antibacterial and antifungal activities against *Bacillus subtilis, Staphylococcus aureus*, *Escherichia coli*, *Pseudomonas aeruginosa*, *Salmonella typhi*, *Candida albicans*, *Penicillium notatum*, *Aspergillus flavus*, *Aspergillus niger* and *Aspergillus solani* were determined. Antioxidant activity was explored using the DPPH radical scavenging method. In vivo analgesic, antipyretic and sedative potential of synthesized nanoparticles was investigated using a mouse model. **Results:** SEM with various magnification powers showed that some particles were spherical while some were aggregated, flake-shaped, and hexagonal with rough and irregular surfaces. The EDX analysis revealed Zn (12.63%), O (22.83%) and C (63.11%) with trace quantities of Si (0.40%), Ca (0.54%) and P (0.49%). The XRD pattern indicated an amorphous state, with no peaks observed throughout the spectrum. The UV–visible spectrophotometry revealed a characteristic absorption peak at 375 nm, indicating the presence of ZnO nanoparticles. Fourier Transform Infrared Spectroscopy (FTIR) displayed several small peaks between 1793 and 2370 cm^−1^, providing evidence of the presence of different kinds of organic compounds with different functional groups. ZnO-NPs showed dose-dependent antibacterial and antifungal potential against all strains. *Staphylococcus aureus* and *Candida albicans* were the most susceptible strains. The nanoparticles exhibited a maximum antioxidant effect of 85.28% at 100 μg/mL. In this study, the acute toxicity test showed no mortality, and normal behavior was observed in mice at ZnO-NP doses of 5, 10, and 20 mg/kg. For analgesic and antipyretic activities, a two-way ANOVA revealed that dose, time, and the interaction between dose and time were significant. In contrast, the samples had a non-significant effect on sedative activity. **Conclusions:** This innovative study suggests a potential use of plant resources for managing microbes and treating various diseases, providing a scientific basis for the traditional use of *H. rariflorum*.

## 1. Introduction

Since antiquity, people have used plants as natural remedies for a wide range of illnesses. The use of herbs is one of the most traditional medical practices, recognized across all civilizations [1]. The World Health Organization (WHO) estimates that 21,000 plants are used in alternative medicine, and over 50,000 plant species are employed in traditional medicine globally. Medicines are derived from the entire plant or various parts, including stems, leaves, bark, roots, flowers, tubers, and seeds, among others. Herbaceous plant species provide protection against a variety of ailments and are considered safe sources of secondary metabolites found in plants [2]. 

Nanotechnology is one of the most versatile fields of recent research, with a wide range of applications. Numerous toxic physicochemical techniques, such as gas-phase methods, spray pyrolysis, electrochemical methods, chemical vapor deposition, and laser ablation techniques, have been familiarized to synthesize nanoparticles [3]. However, these techniques require meticulous process control and involve the use of toxic reagents, expensive instruments, hazardous organic solvents, and non-biodegradable stabilizing agents. As a result, these methods are not only harmful to the environment but also toxic to living organisms [4]. Consequently, there is significant focus on producing nanoparticles (NPs) using simple, safe, and environmentally friendly processes [5]. Plants are more suitable for the fabrication of metal oxide nanoparticles than other sources, due to the abundance of phytochemicals that act as reducing and stabilizing agents, promoting the formation of metallic nanoparticles [6].

Nanoparticles are particulate substances with at least one dimension measuring less than 100 nm [7]. The therapeutic benefits of nanoparticles are influenced by several factors, including the size of the particles, the duration of time the target cells have been in culture, the amount of metal present in the target cells, and their physicochemical characteristics [8]. According to the US Food and Drug Administration (FDA), ZnO is classified as generally recognized as safe. It has numerous practical applications in photocatalysis, biofertilizers, luminescent agents, and biomedical sciences, owing to its high surface area, UV absorption, wide band gap, and greater exciton-binding energy [9]. It possesses cytotoxic, antimicrobial, antioxidant, anticancer, anti-diabetic, anti-inflammatory and anti-aging properties [10].

The use of nanotechnology in the production of effective antimicrobials presents a novel and promising alternative, driven by the need for new agents that can either kill or impede the growth of a wide spectrum of microorganisms [11]. Plant-derived antioxidants are a broad class of naturally occurring compounds with the ability to scavenge radicals, protecting cells from the damage caused by oxidative stress. The antioxidant potential of nanoparticles can be attributed to the presence of phenolic compounds such as flavonoids, tannins, and curcumins [12]. Analgesics are drugs that reduce pain without affecting the patient’s consciousness by acting on the central nervous system or peripheral pain mechanism. Recently, several studies have explored the use of nanotechnology to treat inflammatory pain, with encouraging outcomes [13]. Fever occurs when a person’s body temperature rises above the normal range (36.5–37.5 °C) due to an infection, cancer, or other inflammatory diseases [14]. Drug resistance has made the control and treatment of vectors more challenging. However, many people rely on locally available medicinal plants to treat of pyrexia [15]. Sedatives are central nervous system (CNS) depressants that reduce the activity of specific brain regions, helping to calm or sedate, but their side effects can negatively affect the patient’s quality of life [16]. Nanomedicine, or ‘nanopsychiatry’, utilizes nanoparticles to develop treatments for various neurological and mental disorders [17].

**Figure 1:**
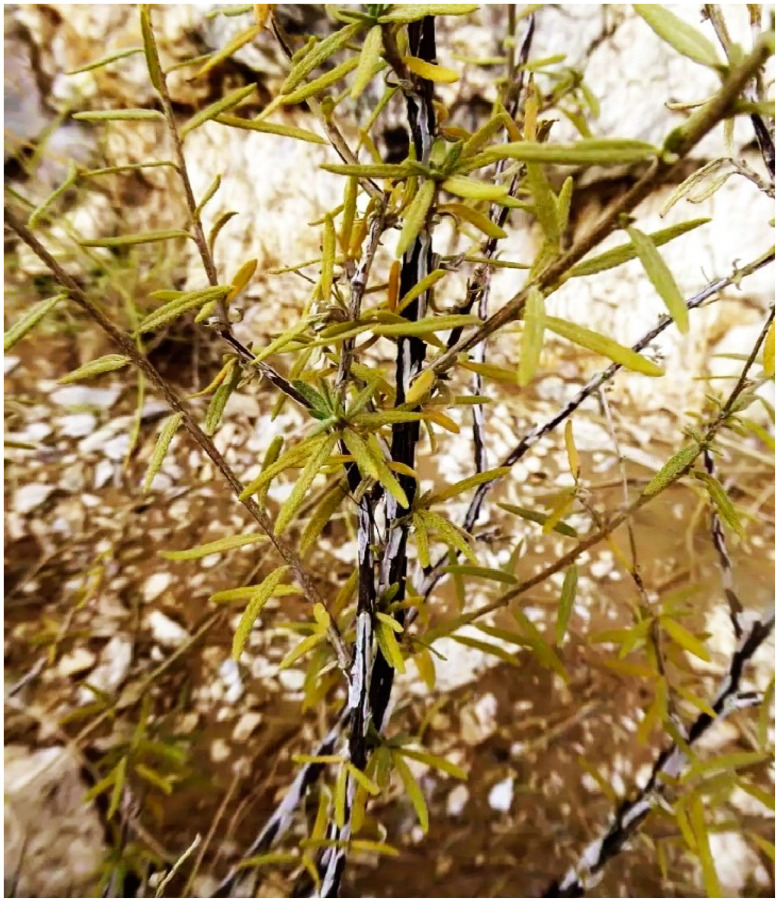
*H. rariflorum* stocks.

*Heliotropium rariflorum* is an erect, spreading, densely branched and hairy perennial herb (Figure 1). The flowering period is from April to July. It is distributed in Iran, Africa, Somalia, Ethiopia, Saudi Arabia, Socotra, Sudan, Oman, Kenya, Yemen, Afghanistan, India and Pakistan. In Pakistan, it is found in Bannu, D. I. Khan, Lakki Marwat and Karak [18]. The different biochemically active constituents that are extracted and characterized from plant species of family Boraginaceae are flavonoids, phenols, pyrrolizidine alkaloids, terpenoids, and naphthoquinones [19]. Some of the plants species of the genus *Heliotropium* are economically important and traditionally practiced and used in folk medicine. However, others show antifungal and antibacterial activities. *H. europaeum*, *H. zeylanicum* and *H. strigosum* show antibacterial and antifungal activities [20]. Moreover, some of the plant species have toxic effects, which cause many liver diseases due to biochemical compounds, i.e., pyrrolizidine alkaloids, the major alkaloids (Helifoline and Retronecine) in *H. ovalifolium,* which are hepatotoxic [21].

Keeping in view the antimicrobial, antioxidant, analgesic, sedative, and antipyretic activities [22,23,24,25,26,27] of other species of *Heliotropium* (*Heliotropium indicum*, *Heliotropium strigosum*, *Heliotropium bacciferum*, *Heliotropium subulatum*) and the growing importance of *H. rariflorum* in the pharmaceutical industry, which has not been previously addressed, it was hypothesized that *H. rariflorum* might also have such activities. Moreover, the growing interest in the green synthesis of ZnO-NPs, along with its biomedical applications in various fields, and its core properties of eco-friendliness and cost-effectiveness made it an attractive choice to explore. Therefore, the present study was conducted to evaluate the in vitro and in vivo pharmacological properties of *H. rariflorum*-mediated ZnO-NPs and their potential in different metabolic pathways, as well as practical medical applications that were presented.

## 2. Results and Discussion

### 2.1. Characterization of ZnO-NPs

#### 2.1.1. SEM

**Figure 2:**
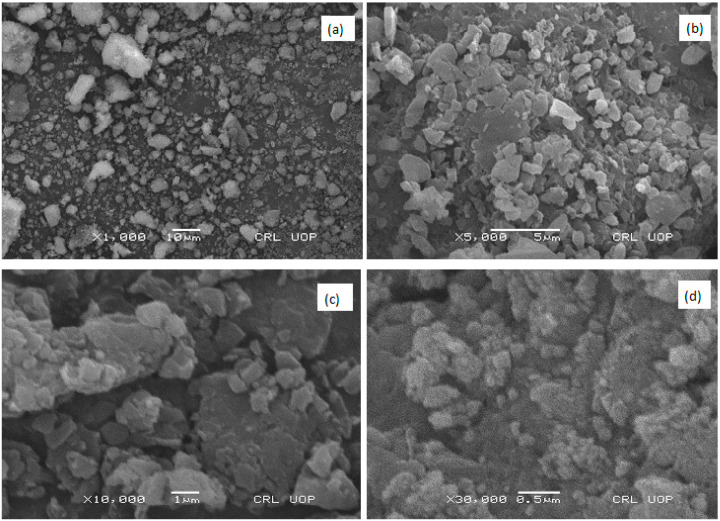
SEM of the ZnO-NPs with magnification power of (**a**) 1000× at 10 μm, (**b**) 5000× at 5 μm, (**c**) 10,000× at 1 μm, and (**d**) 30,000× at 0.5 μm.

**Figure 3:**
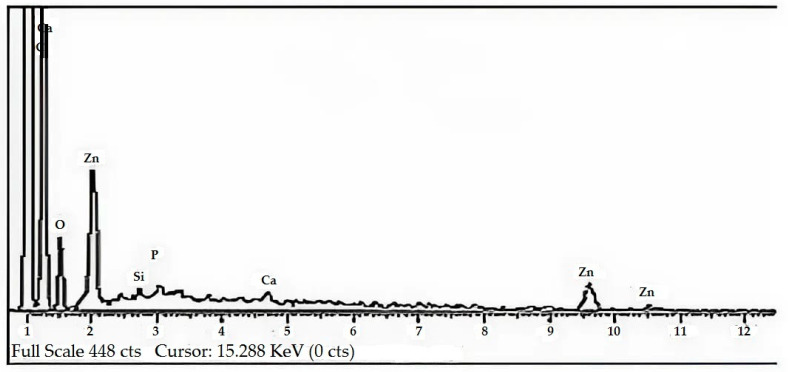
EDX analysis of the ZnO-NPs.

SEM of the zinc oxide nanoparticles with various magnification powers, i.e., 1000× at 10 μm bar length (Figure 2a), 5000× at 5 μm bar length (Figure 2b), 10,000× at 1 μm bar length (Figure 2c), and 30,000× at 0.5 μm bar length (Figure 2d), was carried out to study the morphology. The shape of the particles was not uniform. Most of them were spherical while some were flake-shaped and hexagonal with rough and irregular surfaces. An aggregation of the nanoparticles was seen in SEM (Figure 2). This does not disturb the curative properties of biosynthesized ZnO-NPs. The smaller sized nanoparticles bind together and form agglomerated nanoparticles. The small size enhances the Zn^2+^ dissolution rate, making it more biocompatible with body cells and a potential therapeutic agent [28,29]. Such kind of agglomeration can also be detected in other reports on plant-mediated ZnO-NPs [30,31]. The average size was determined to be in the range of 80 to 90 nm (Figure 3). Similar spherical and hexagonal plant-mediated, highly aggregated ZnO-NPs with rough surfaces were reported in early studies [32]. The form of nanoparticles (NPs) is a critical factor in their efficacy against infections. For example, spherical NPs are often highly effective in terms of antibacterial activity because they can readily pierce pathogen cell walls [33]. The agglomeration of NPs occurs due to the presence of various functional groups (FTIR) and maximum reduction [34]. According to Murali et al. [35], a calcination step is obligatory to achieve pure ZnO-NPs and remove organic matter from the plant extract. In this study, it was seen that calcination (400 °C) did not remove all organic matter from the synthesized NPs and converted the phenolic compounds capping the ZnO-NPs into amorphous carbon, which caused agglomeration and particle coarsening (Figure 2).

#### 2.1.2. EDX Analysis

The EDX spectrum confirmed the synthesis of ZnO-NPs showing well-defined peaks at 1, 8.5, and 9.5 keV. Some other peaks were also seen in the spectrum, i.e., phosphorus (P) at 2 keV, silicon (Si) at 1.8 keV, carbon (C) at 0.3 keV, calcium (Ca) at 0.2 and 3.7 keV, and oxygen (O) at 0.5 keV. The elemental composition revealed Zn (12.63%), O (22.83%), and C (63.11%) with trace quantities of Si (0.40%), Ca (0.54%), and P (0.49%). Since the nanoparticles were biogenic and obtained from *H. rariflorum*, C, Si, Ca, and P were also observed in the spectrum. Furthermore, both Zn and O were homogeneously found throughout the sample (Figure 3). The current findings are consistent with the published literature [8,36,37].

#### 2.1.3. XRD Analysis

**Figure 4:**
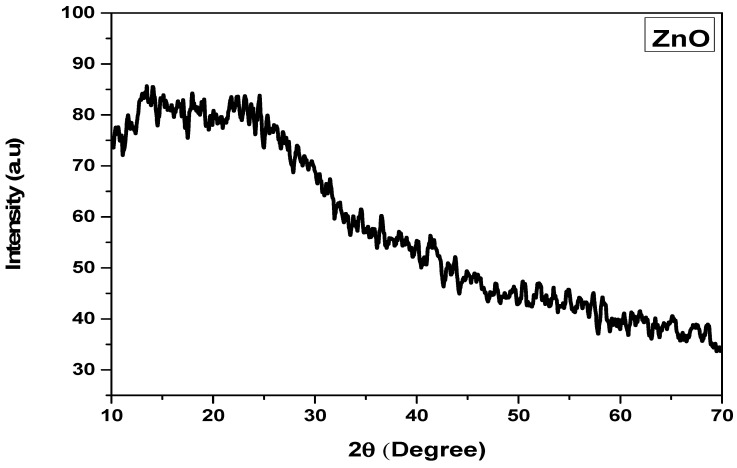
XRD analysis of the ZnO-NPs.

The X-ray diffraction spectrometry of ZnO-NPs was performed to obtain insight into their structural properties. The XRD pattern revealed an amorphous state. The spectrum showed no peaks as evidence for the amorphous nature of zinc oxide nanoparticles. As amorphous forms have no specific geometry, no further attempts can be made to determine their structural behaviors. The amorphous form can be associated with the biological origin of the particles as the nanomaterial was extracted from *H. rariflorum* (Figure 4). Samy et al. [38] investigated the X-ray diffraction of bio-extracted ZnO-NPs and reported amorphous structure. According to Menazea et al. [39], biosynthesized ZnO-NPs have less crystal structure compared to chemically synthesized particles because of the difference in the arrangement of atoms inside the particles. Bala et al. [40] observed similar amorphous *Hibiscus subdariffa*-mediated zinc oxide nanoparticles in their study. Amorphous nanoparticles have the benefit of synergistic mechanisms that increase supersaturation levels and enhance dissolution rates in the biomedical area. One example of this technique is NanomorphTM, which was created by Soliqus/Abbott [41,42,43].

#### 2.1.4. UV–Visible Spectrophotometry

**Figure 5:**
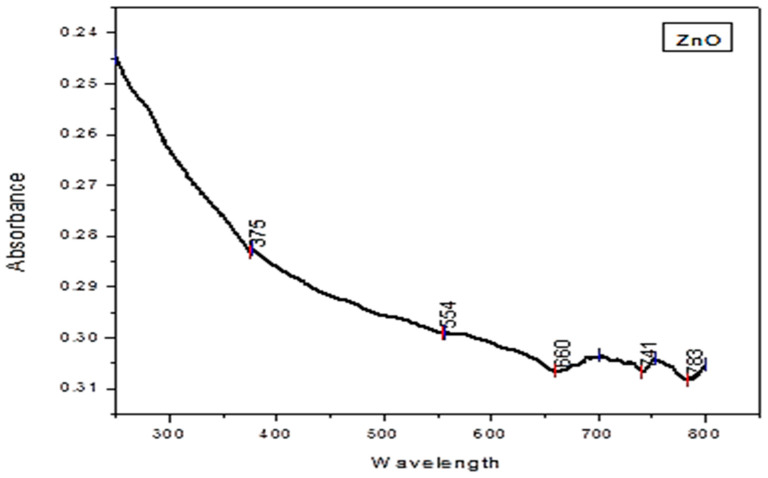
UV–visible spectrophotometry of ZnO-NPs.

The UV–visible spectrophotometry of *H. rariflorum*-mediated zinc oxide nanoparticles was recorded in a range of 200–800 nm. It showed a characteristic absorption peak at 375 nm indicating the presence of ZnO nanoparticles. The bandgap energy (3.3 eV) of synthesized nanoparticles was determined using Eg = 1240/λ eV, which is analogous to the published literature [44,45,46]. ZnO is a II–VI semiconductor, where Zn and O are found in groups 2 and 6 on the periodic table, respectively [47]. The high bandgap energy (3.3 eV) makes it a potential conducting material to be used in optoelectronic devices, electronics and photocatalysis [48]. Some other peaks were also observed at 554, 660, 741, and 783 nm, which may be due to the presence of other compounds as the zinc oxide nanoparticle samples were biosynthesized and may contain several other compounds as well (Figure 5). MuthuKathija et al. [49] characterized *Pisonia alba*-mediated ZnO-NPs through UV-Vis spectra and reported a maximum peak at 378 nm. Their results are in line with the present study. Mohammed et al. [50] and Rajeshkumar et al. [51] documented peaks in the same region. Rasheed et al. [52] claimed that flavonoids convert the metallic ions into nanoparticles by donating a responsive hydrogen atom through the shifting of their OH groups from the enol mold to the keto mold. They have great sensitivity to the metals to reduce Zn^2+^ to Zn^0^ [53]. Throughout the process, the resulting Zn^0^ is oxidized to ZnO-NPs [54].

#### 2.1.5. FTIR

**Figure 6:**
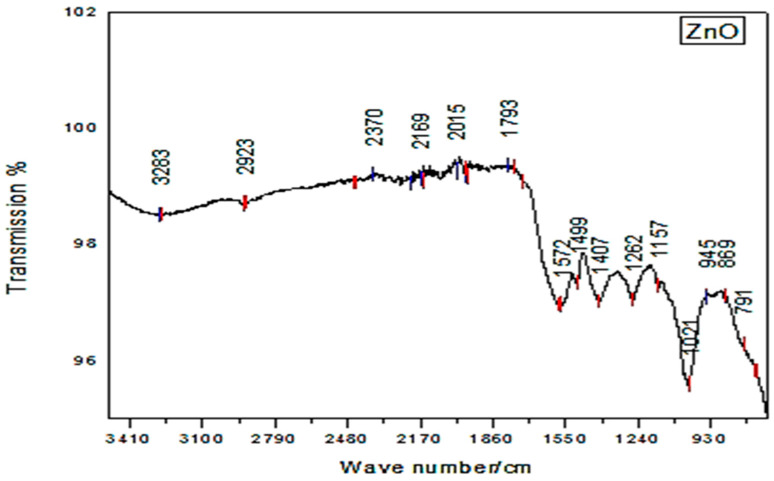
FTIR spectrum of ZnO-NPs.

The identification of various compounds in the biosynthesized ZnO-NPs was performed by analyzing the nanoparticles with Fourier Transform Infrared Spectroscopy (FTIR) in the range of 400–4500 cm^−1^. It showed various small peaks from 1793 to 2370 cm^−1^, providing evidence of the presence of different kinds of organic compounds with different functional groups. The peak at 2923 cm^−1^ revealed the presence of –CH_2_-, while a broad peak at 3283 cm^−1^ helped to identify the presence of O-H bond vibration of alcohols and phenol. The absorption peaks, which were comparatively sharp and show higher intensity, can be observed in the FTIR spectra ranging from 1021 to 1572 cm^−1^. Among these prominent peaks, 1572 cm^−1^ was considered to represent zwitterions of amino acids; 1407 cm^−1^ might be accredited as evidence of C=O bonding; 1262 cm^−1^ is attributed to carboxylic acids; and the one at 1121 cm^−1^ highlights the aliphatic ethers that occupy this place. The peaks at 869 cm^−1^ and 791 cm^−1^ are attributed to the formation of ZnO-NPs (Figure 6). These results suggested that the OH groups of flavonoids, the protein molecules, and their functional groups played a crucial role in the bioreduction of the salt and the capping of NPs [55]. Yang et al. [56] and Dastidar et al. [57] also agreed with the current results. These functional groups converted Zn^+2^ to Zn and resulted in the biosynthesis of NPs [33].

### 2.2. In Vitro Biological Properties

#### 2.2.1. Antibacterial Activity

**Figure 7:**
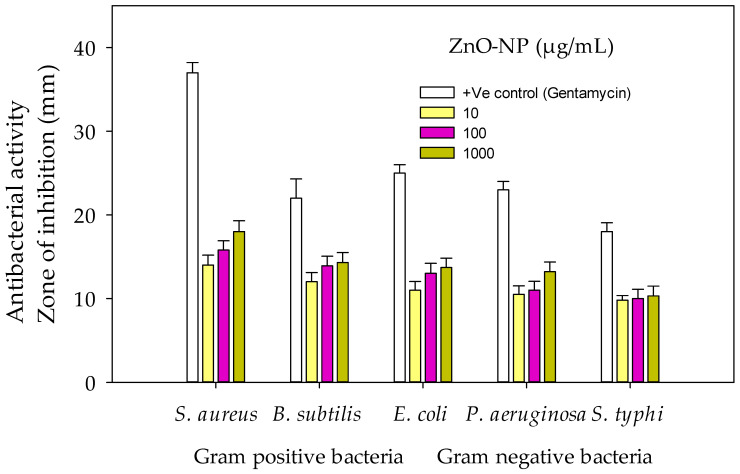
Antibacterial activity of ZnO-NPs.

**Figure 8:**
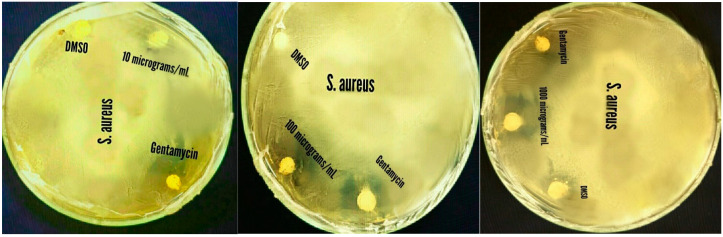
Growth inhibition of *Staphylococcus aureus* at 10, 100 and 1000 µg/mL of ZnO-NPs.

The results showed that ZnO-NPs caused maximum (18 and 15.8 mm) growth inhibition of *Staphylococcus aureus* at 1000 and 100 µg/mL. The least inhibition (9.8 mm) of *Salmonella typhi* was shown at 10 µg/mL. The effect was dose-dependent, and it increased with the increase in dose level. Furthermore, it was revealed that Gram-positive bacteria (*Bacillus subtilis* and *Staphylococcus aureus*) were susceptible as compared to Gram-negative bacteria *(Escherichia coli*, *Pseudomonas aeruginosa* and *Salmonella typhi).* However, the antibacterial effect of nanoparticles was greater than the blank (0 mm) and less than Gentamycin (standard drug) against all tested bacteria (Figure 7 and Figure 8).

Many researchers like Mwafy et al. [58] and Fouda et al. [59] thoroughly explored the antibacterial potential of green synthesized ZnO-NPs and the results are consistent with the current study. ZnO-NPs cause cell death through direct injury to cells or indirect generation of ROS to inhibit protein function and produce harmful lipid peroxidation in bacteria [60]. The potential of these NPs was due to the proteins contained in the NPs. Proteins anchor to the cells of bacteria by LysM domains and bind to the peptidoglycan in the cell walls, causing lysis of the cells [61]. The present results are in similarity with those presented by Velsankar et al. [62] who reported that Gram-negative strains were comparatively resistant. The size of the particles, the temperature at which the nanoparticles are synthesized, the composition of the bacterial cell wall, and the degree of interaction between the nanoparticles and bacteria are the variables that influence the sensitivity of bacteria to zinc oxide nanoparticles [63]. Gram-negative bacteria have a selectively permeable membrane, are less predisposed to NPs, secrete enzymes β-lactamases, destroy the administered antibiotics, and thus offer strong resistance [64].

#### 2.2.2. Antifungal Activity

**Figure 9:**
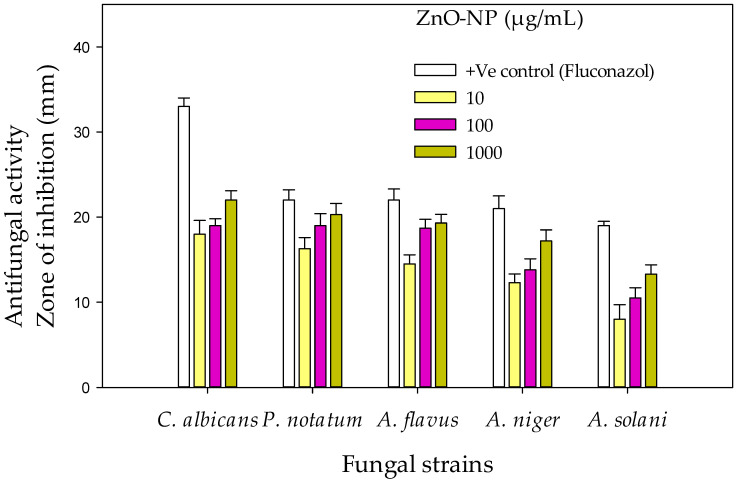
Antifungal activity of ZnO-NPs.

**Figure 10:**
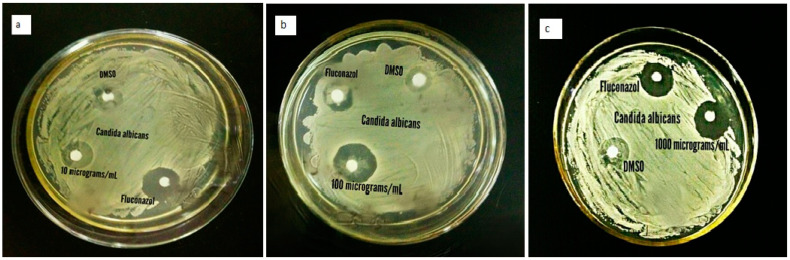
Growth inhibition of *Candida albicans* at (**a**) 10, (**b**) 100 and (**c**) 1000 µg/mL of ZnO-NPs.

ZnO-NPs revealed maximum inhibition (22, 19 and 18 mm) of *C. albicans* at 1000, 100 and 10 µg/mL, respectively. The minimum zone of inhibition (8 mm) was shown against *Alternaria solani* at 10 µg/mL. In the current study, the activity was lower than fluconazole (33 mm) but higher than the blank (0 mm). The effect was concentration-dependent. It was noted that *Candida albicans* was the most susceptible fungus as compared to the rest of the test fungi (Figure 9 and Figure 10). Sharma et al. [65] and Kamal et al. [66] reported similar findings. NPs induce changes in protein expression, which is the key to inhibiting microbial growth or oxidative stress, leading to cell death [67]. These compounds enter the cell membrane of a fungus and cease cell division via strong interactions on the respiratory chains [68].

#### 2.2.3. Antioxidant Activity

Antioxidants are materials that prevent cellular impairment induced by reactive oxygen species (ROS). They play a vital role in scavenging free radicals and precluding oxidative damage [69]. In the present study, ZnO-NPs possessed dose-dependent activity. It had the maximum antioxidant effect (85.28%) at 100 μg/mL. Minimum activity (45%) was shown at 5 μg/mL when compared to the blank (0%). However, the potential was lower than that of ascorbic acid (97.45%) at the same dose levels. Furthermore, the NPs’ IC_50_ value was 12.085 µg/mL compared to that of ascorbic acid, 0.0079 µg/mL (Table 1). Awan et al. [33] worked on *Ailanthus altissima*-mediated ZnO-NPs and reported dose-dependent antioxidant activity, with an IC_50_ value of 78.23 µg/mL, higher than that of ascorbic acid (61.75 µg/mL). The lower the IC_50_ value of the sample, the greater the antioxidant potential [70]. According to Ahani et al. [71], total phenolic contents (TPCs) and antioxidant activity have a positive relationship, so the highest activity of the nanoparticles was due to maximum contents of phenolic compounds in the studied plant. The redox features of TPC contribute to their antioxidant potential by acting as reducing agents, hydrogen donors, metal chelators, and oxygen quenchers. The –OH groups in phenolic compounds react with ROS, breaking the process of ROS formation [72]. High-potential medicinal plants can be used as a natural source of antioxidants to treat a variety of oxidative stress-related conditions, such as cancer and certain heart conditions [73].

### 2.3. Acute Toxicity Testing

No mortality was noticed during the acute toxicity test at any dose (5, 10, 20 mg/kg) over 24 h. The behavior of the mice was normal throughout the experimentation. No mice showed any signs of toxicity; their behavior was normal even after 36 h (Table 2). In toxicology and pharmacology, acute toxicity has been extensively used as a behavioral assay [74]. In previous studies, ZnO-NPs showed toxicity at relatively high doses treated by oral administration [75]. Sharma et al. [76] reported toxicity in mice at 300 mg/kg dose, but not at 50 mg/kg. Other modes of administration through skin or inhalation displayed different results [77]. Xu et al. [78] claimed that acute toxicity in mouse models depends on the mode of administration of the ZnO-NPs. Djouadi and Derouiche [79] evaluated ZnO-NPs for toxicity at doses of 70 mg/kg and below, but no toxic signs were reported at all doses.

### 2.4. In Vivo Biological Properties

#### 2.4.1. Analgesic Activity

The results revealed the highest pain reduction (91%, 88%, and 86.25%) at 20, 10, and 5 mg/kg, respectively, as compared to the control (0%) and standard drug tramadol (81%). Two-way ANOVA revealed that doses, time, and dose and time interaction were statistically significant (*p* < 0.01), i.e., *p* = 0.000 (Table 3). The release of endogenous mediators from tissue phospholipids, such as arachidonic acid, is triggered by acetic acid and results in the synthesis of prostaglandin and pain [73]. Other workers, especially Devientasaria et al. [80], investigated *Vernonia amygdalina*-, *Euphorbia milii*-, and *Abies spectabilis*-mediated ZnO-NPs and reported significant results as compared to controls. The analgesic effect of nanoparticles and metabolites is related to enzymes and receptors including cyclo-oxygenase, gamma-aminobutyric acid (GABA), and ionic channel and opioid receptors, thus inhibiting the synthesis of prostaglandins and ultimately reducing pain sensations [81].

#### 2.4.2. Antipyretic Activity

Results showed that the subcutaneous yeast injection resulted an increase in rectal temperature, which peaked at 38.174 °C. The administration of NPs caused a decrease in pyrexia. ZnO-NPs reduced it to 36.03 °C and 36.1 °C at 20 and 10 mg/kg. Two-way ANOVA revealed that doses, time, and dose and time interaction were highly significant (*p* = 0.000) (Table 4). Rauf et al. [82] investigated the antipyretic potential of *Lentinula edodes*-mediated ZnO-NPs and reported that the nanoparticles possessed highly significant effects compared to controls and standard drugs. Tannins, steroids, saponins, and flavonoids are major inhibitors of cyclooxygenase and lipoxygenase, lower prostaglandin levels, and thus lead to a reduction in fever [83]. Nanoparticles may also have an antipyretic effect through mediating superficial blood vessel dilatation, which enhances heat loss through the hypothalamus thermostat reset [84]. Synthetic antipyretic drugs reduce increased body temperature but they also pose a risk to the kidneys, heart, liver, and brain [85]. On the other hand, metal oxide nanoparticles are currently extensively employed in pharmaceuticals because of their valuable qualities, such as great stability, biocompatibility, lack of toxicity, and precise aggregation actions towards target cells and tissues [86].

#### 2.4.3. Sedative Activity

The results of the study exhibited significant dose-dependent suppression of movement in 10 min. The NPs possessed the highest movement inhibition (82%, 70%, and 66%) at 20, 10, and 5 mg/kg compared to the control, but to a lesser extent than the standard drug diazepam (96%). One-way ANOVA displayed a non-significant effect of the samples (*p* = 0.259) (Table 5). Ajaykumar et al. [87] and Wang et al. [88] worked on plant-mediated ZnO-NPs and observed a high percentage of activity as compared to controls, but less than the standard drugs diazepam and morphine. But ANOVA demonstrated a mild effect. NPs exhibit anxiolytic and sedative activity through its interactions with GABA receptors. They enhance the biological properties of plant extracts, promoting the release of bioactive metabolites and decreasing side effects [89]. Alkaloids, flavonoids, steroids, terpenoids, and saponins contained in the crude extracts and NPs bind to the central benzodiazepine receptors, increase chloride ion conductance, decrease activity, moderate excitement and calm the recipient, and maintain sedation [81]. Synthetic drugs reduce anxiety and have a calming effect. The long-term use of these medications frequently results in serious side effects, such as immunological system, lung, and gastrointestinal dysfunction as well as neurocognitive impairment, physical dependence, and addiction. Therefore, in order to manage a variety of mental illnesses, it has been advised to develop novel sedative drugs on nanoscales from plants with less side effects [90,91].

## 3. Materials and Methods

### 3.1. Plant Collection, Identification, and Grinding

Fresh samples of *H. rariflorum* were collected from District Karak, Pakistan. They were identified with the help of a plant list of the flora of Pakistan [18,92]; voucher number Noor Ul Uza Bot. 01. (PUP) was assigned and deposited in the Herbarium of the Department of Botany, University of Peshawar, Pakistan. The plants were shade-dried and pulverized into fine powder using an electric grinder [93]. The powder was extracted in distilled water and used for bio-fabrication of ZnO-NPs.

### 3.2. Green Synthesis of Nanoparticles

**Figure 11:**
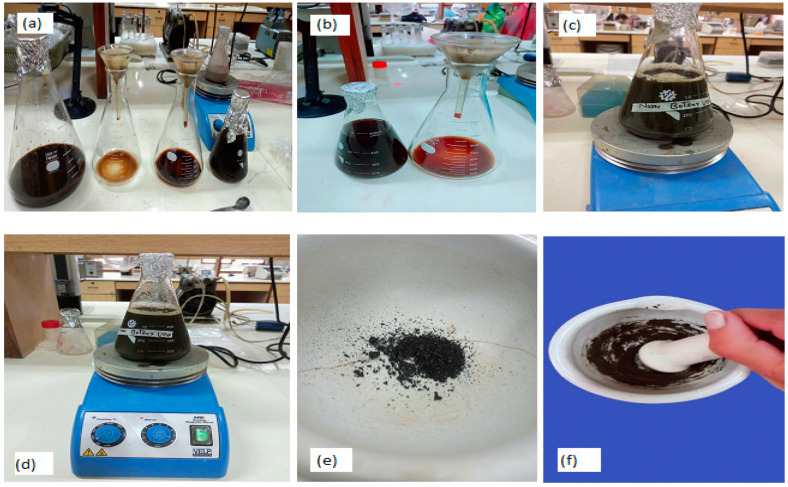
Green synthesis of *H. rariflorum*-mediated ZnO-NPs. (**a**,**b**) filtration of plant extract (**c**,**d**) stirring of plant extract on magnetic stirrer after salt addition (**e**) synthesis of ZnO nanoparticles (**f**) crushing of ZnO nanoparticles.

ZnO-NPs were synthesized using the procedure of Basit et al. [94] at the Institute of Biotechnology Genetic Engineering (IBGE), the University of Agriculture, Peshawar. Zinc acetate dihydrate (Zn(CH_3_COO)_2_·2H_2_O) was dissolved in distilled water to prepare a salt solution (0.2 M = 43.9 g/L). Then, 50 mL of salt solution was added to plant aqueous extract (20 mL) and stirred via an electric stirrer at 80 °C and 700 rpm. In order to maintain the pH at 13, NaOH was added until the formation of bright peach-colored precipitates. The samples were filtered with muslin cloth and Whatman filter paper # 1. The reaction mixture was again stirred for 2 h and the precipitates were accumulated. The precipitates were moved to Eppendorf tubes, and the supernatant was disposed of. The samples were centrifuged at 6000 rpm for 15 min and washed with distilled water 3–4 times to obtain pellets. Calcination of these pellets was carried out at 400 °C for 3 h. The resultant sample was transformed into a homogeneous powder (Figure 11).

### 3.3. Characterization of Synthesized Nanoparticles

Characterization (SEM, EDX, XRD, UV-Vis and FTIR) of bio-fabricated ZnO-NPs was performed according to the protocol of Dua et al. [95] at CRL, Department of Physics, University of Peshawar, Pakistan.

#### 3.3.1. Surface Morphology Analysis (SEM)

SEM of the nanoparticles was performed to study the surface morphology using a Scanning Electron Microscope (Model JEOL-JSM-5910). The samples were prepared by keeping the synthesized nanoparticles above the carbon-coated stub; excess nanoparticles were removed with an air dust blower. The particles were prepared for imaging without coating. The voltage was maintained at 15 kV and magnification was maintained at 1000, 5000, 10,000 and 30,000×. Moreover, the size of the synthesized ZnO-NPs was measured in nanometers using statistical software, Nano Measurer 1.2.5.

#### 3.3.2. Energy-Dispersive X-Ray Analysis (EDX)

The elemental composition of nanoparticles was evaluated using Scanning Electron Microscopy (SEM) coupled with an Energy-Dispersive X-ray (EDX) analyzer (JEOL, JSM-6380 LA, Tokyo, Japan).

#### 3.3.3. XRD

The crystalline structure of nanoparticles was investigated with the help of an X-ray diffractometer (JEOL 5910), using 40 Kv and 40 mA current, with Co-Kα radiation (*λ* 1.5405 A°). The spectra were recorded between 30° and 80° (2-*θ* range). The findings were appraised using the Joint Committee on Powder Diffraction Standards (JCPDS) library to determine the crystalline structure. The particle size was calculated using the equation of Debye–Scherrer.

$$D=\frac{K\lambda }{\beta cos\theta }$$

where

*D* = grain lattice size;

*λ* = 1.5406 A°;

β = full width at half maximum (FWHM);

*θ* = Bragger’s angle of diffraction.

#### 3.3.4. UV-Vis Spectroscopy

A 100 µg/mL aqueous suspension of synthesized nanoparticles was prepared and homogenized in a sonicator for 5 min. Water was used as a blank. The test sample was fixed in a spectrophotometer (Shimadzu, Japan). UV light was passed at 300 to 700 nm and confirmed the synthesis of nanoparticles.

#### 3.3.5. FTIR Spectroscopy

Fourier Transform Infrared Spectroscopy (FTIR) was carried out to identify the functional groups involved in the green synthesis of nanoparticles. The suspension was desiccated at 75 °C and characterization was performed at a wavenumber ranging from 4000 to 400 cm^−1^ using a Perkin Elmer FTIR spectrometer, spectrum-2, equipped with UATR-2.

### 3.4. In Vitro Biological Properties of Biosynthesized ZnO-NPs

#### 3.4.1. Antibacterial Activity

Antibacterial activity of ZnO-NPs against *Staphylococcus aureus, Bacillus subtilis,* (Gram-positive), *Pseudomonas aeruginosa, Escherichia coli,* and *Salmonella typhi* (Gram-negative) was determined as explained by Takele et al. [96]. For bacterial culturing, 15 g of agar powder was dissolved in 1000 mL of distilled water and autoclaved for 15 min at 121 °C. The bacterial culture (10 mL) was added to agar and incubated at 30 °C for 24 h. Stock solution (20 mg mL^−1^) of nanoparticles was prepared in 1% dimethyl sulfoxide (DMSO). Gentamycin and DMSO (1%) were used as positive and negative controls, respectively. Wells were made in the medium; stock solution of 10, 100 and 1000 µgmL^−1^ was supplied and incubated for 24 h at 37 °C. The zones of growth inhibition (mm) were measured.

#### 3.4.2. Antifungal Activity

Antifungal activity of NPs against *Candida albicans*, *Penicillium notatum*, *Aspergillus flavus*, *Aspergillus niger,* and *Aspergillus solani* was assessed using the method of Ameen et al. [97]. Sabouraud Dextrose Agar (SDA) was prepared, autoclaved, poured into sterile Petri plates, and incubated at 28 °C for 24 h. Stock solution was prepared for test samples at a concentration of 20 mg/mL in DMSO. The SDA, when heated to 50 °C, in 10, 100, and 1000 µg/mL stock solution, was added to Petri dishes. Old fungal strains were cultured on solidified media and were incubated for 7 days at 28 °C. DMSO and fluconazole served as negative and positive controls, respectively. Zones of inhibition (mm) were measured.

#### 3.4.3. Antioxidant Activity

DPPH solution (0.2 mM) was prepared in methanol and stirred overnight at 4 °C. The various dose levels (5, 10, 20, 30, 40, 60, 80, and 100 μg/mL) of the samples (0.5 mL) were combined with 2 mL of DPPH (0.2 mM) in each flask. To prevent light from shining on the solution, aluminum foil was used to cover it, and it was incubated in a dark environment for 30 min. The absorbance of the blank solution (2 mL of DPPH) was documented using UV-Vis spectroscopy. After the incubation period, each test solution was observed for its absorbance at 517 nm, as DPPH reveals a strong absorption band at this wavelength that vanishes following electron pairing, causing a loss of the deep violet color. Ascorbic acid and methanol were used as positive and negative controls, respectively. The following formula was used to calculate the % DPPH scavenging activity of the test samples [98]:

$$\%\;DPPH\;scavenging\;activity=\frac{Ab-As}{Ab}\times 100$$

where

Ab = absorbance of negative control;

As = absorbance of sample solution.

### 3.5. Acute Toxicity Testing

The acute toxicity test of the ZnO-NPs was evaluated using Lorke’s method [99]. Albino mice (aged 4–5 weeks) of average weight (25 gm) were collected from the Veterinary Research Institute (VRI), KP, Peshawar, and were kept under accustomed laboratory conditions, i.e., relative humidity of 55%, temperature of 23 ± 2 °C, and a 12:12 h light/dark cycle, with a light intensity of 150–300 lux. They were provided a standard diet and water ad libitum under stern aseptic conditions. The procedure was approved by the Ethical Review Board, Department of Biotechnology, University of Science and Technology, KP (Ref. No. USTB\ethic/112). Twenty mice were categorized into four groups (n = 5); all animals were kept fasting for 12 h. Three doses of nanoparticles (5, 10, and 50 mg/kg body weight) were chosen for the test. Group I was treated with control, while groups II, IIIs and IV were treated with ZnO-NPs at 5, 10, and 50 mg/kg body weight concentration, respectively. The animals were continuously checked for 24 h to screen their behavior and mortality, if any. Then, mice were kept under observation for 20 days to observe their mortality. This observation was made twice a day.

### 3.6. In Vivo Pharmacological Evaluation of ZnO-NPs

For in vivo pharmacological activities, albino mice (aged 4–5 weeks) of average weight (25 gm) were collected from the Veterinary Research Institute (VRI), KP, Peshawar. They were kept at 55–65% relative humidity and 24 °C temperature for 7 days. Mice were fed VRI formulated food. For study, animals were divided into 5 groups, each having 5 mice. Three doses (5, 10, 20 mg/kg) were selected on the basis of acute toxicity testing. The protocols were approved by the Ethical Review Board, Department of Biotechnology, University of Science and Technology, KP (Ref. No. USTB\ethic/112).

#### 3.6.1. Analgesic Activity

Animals were treated with test samples intra-peritoneally. Mice in group I received normal saline (10 mL/kg) and served as negative controls, while group II mice were administered with tramadol (30 mg/kg) and served as positive controls. Likewise, groups III, IV, and V were treated with ZnO at dose levels of 5, 10, and 20 mg/kg; 1% of acetic acid was administered after 30 min of therapy [100]. After 5 min of injection, the number of writhing actions was counted for next 15 min.

$$\%\;Pain\;inhibition=\frac{Mean\;writhes\;of\;negative\;control-mean\;writhes\;of\;test\;group}{Mean\;writhes\;of\;negative\;control}\times 100$$

#### 3.6.2. Antipyretic Activity

The animals were fasted for 12 h and their initial body temperature was checked before the treatment of yeast solution (20%). To induce pyrexia, 10 mL/kg of yeast solution was i. p. injected into mice. Animals in group I received normal saline (10 mL/kg) and served as negative controls, while group II was administered with 20 mg/kg of paracetamol and served as the positive control. Groups III, IV, and V were treated with ZnO-NPs at dose levels of 5, 10, and 20 mg/kg. The temperature was measured for 4 h [101].

#### 3.6.3. Sedative Activity

Animals in group I received normal saline (10 mL/kg) and served as negative controls, while group II was administered with diazepam (0.5 mg/kg) and served as the positive control. Groups III, IV, and V were treated with ZnO-NPs at dose levels of 5, 10, and 20 mg/kg intra-peritoneally. The apparatus used in this study was made of stainless steel and allocated into 19 squares. After 30 min, each animal was placed in the center of box and the numbers of lines crossed were counted for each mouse for 10 min. A smaller number of lines crossed indicated a sedative effect [99]. The percent movement inhibition was calculated as

$$\%\;Movements\;inhibition=\frac{Mean\;number\;of\;movements\;in\;negative\;control-Mean\;number\;of\;movements\;in\;test\;groups}{Mean\;number\;of\;movements\;in\;negative\;control}\times 100$$

### 3.7. Statistical Analysis

All data of the in vitro (n = 3) and in vivo bioassays (n = 5) were expressed as mean ± SEM. For antioxidant activity, IC_50_ values were calculated from the % inhibition versus concentration by a sigmoidal curve using a non-linear regression analysis. One-way (sedative activity) and two-way (analgesic and antipyretic) analyses of variance (ANOVAs) were performed using the Costat Software Computer Program accompanied by a post hoc Least Significance Difference (LSD) test between groups at *p* < 0.05.

## 4. Conclusions and Recommendations

In the current study, *H. rariflorum*-mediated ZnO-NPs were synthesized and characterized through SEM, EDX, XRD, UV, and FTIR. SEM revealed aggregated nanoparticles in various shapes with 80–90 nm size. The EDX spectrum revealed the fabrication of ZnO-NPs displaying peaks at 1, 8.5, and 9.5 keV. It showed Zn (12.63%), O (22.83%), C (63.11%), Si (0.40%), Ca (0.54%), and P (0.49%). The XRD showed an amorphous state with no peaks in the spectrum. UV–visible spectrophotometry showed a characteristic absorption peak at 375 nm indicating the synthesis of nanoparticles. Other peaks were seen at 554, 660, 741, and 783 nm, which were attributed to the presence of phytochemicals in plant extracts. FTIR showed various peaks from 1793 to 2370 cm^−1^ providing evidence of synthesized nanoparticles. For the identification of various secondary metabolites in the biogenic ZnO-NPs, Fourier Transform Infrared Spectroscopy (FTIR) was performed in the range of 400–4500 cm^−1^. It demonstrated various peaks from 1793 to 2370 cm^−1^ providing evidence of the existence of organic compounds with different functional groups. Antibacterial and antifungal activities of synthesized nanoparticles were determined using *Bacillus subtilis*, *Staphylococcus aureus* (Gram-positive), *Escherichia coli*, *Pseudomonas aeruginosa* and *Salmonella typhi* (Gram-negative), *Penicillium notatum*, *Candida albicans*, *Aspergillus flavus*, *Aspergillus solani* and *Aspergillus niger*. Activities were concentration-dependent. *S. aureus* and *C. albicans* were the most susceptible microbes in this study. ZnO-NPs possessed good antioxidant potential (IC_50_ = 12.085 µg/mL) as compared to blanks. Prior to in vivo pharmacological activities, NPs were tested for acute toxicity at doses of 5, 10, and 20 mg/kg. No toxicity and mortality were reported at said doses. Therefore, these doses were chosen for pharmacological evaluation of ZnO-NPs. They displayed noteworthy in vivo pharmacological activities, i.e., analgesic and antipyretic, at all doses. On the other hand, the in vivo sedative was statistically non-significant. Consequently, the suggested procedure is simple, safe, and non-toxic. It is recommended that ZnO-NPs have the potential to be used as antimicrobial, antioxidant, analgesic, antipyretic, and sedative agents. They will provide cost-effective and potent drugs for the treatment of various ailments.

## Figures and Tables

**Table 1:** Antioxidant activity of *H. rariflorum* stocks.

S. NO	Concentration (μg/mL)	Ascorbic Acid	Blank	ZnO-NPs
		(%) Free Radical Scavenging		
1	5	90.30	0	45
2	10	92.15		48.2
3	20	95		50.21
4	30	95.17		55.44
5	40	95.22		59.13
6	60	96		79.34
7	80	97.40		80.30
8	100	97.45		85.28
IC_50_ values		0.0079		12.085

**Table 2:** Acute toxicity test of *H. rariflorum*-assisted ZnO-NPs.

Behavior Observations	ZnO-NPs (mg/kg)	Duration					
Mortality	Control	0 h	2 h	4 h	**8 h**	**24 h**	**36 h**
	5	None	None	None	None	None	None
	10	None	None	None	None	None	None
	20	None	None	None	None	None	None
Feed and water consumption	5	Normal	Normal	Normal	Normal	Normal	Normal
	10	Normal	Normal	Normal	Normal	Normal	Normal
	20	Normal	Normal	Normal	Normal	Normal	Normal
Diarrhea	5	None	None	None	None	None	None
	10	None	None	None	None	None	None
	20	None	None	None	None	None	None
Movement	5	Normal	Normal	Normal	Normal	Normal	Normal
	10	Normal	Normal	Normal	Normal	Normal	Normal
	20	Normal	Normal	Normal	Normal	Normal	Normal

**Table 3:** In vivo analgesic activity of *H. rariflorum* stocks.

Treatments	Dose (mg/kg) Body Weight	Writhing (Mean ± SEM)			% Inhibition
		After 5 min	After 10 min	After 15 min	
Normal saline (10 mL/kg)		33.00 ± 0.76	33.00 ± 0.76	32.00 ± 0.93	0
Tramadol	30	15.20 ± 0.67	14.00 ± 0.44	6.00 ± 0.44	81
ZnO-NPs	5	11.60 ± 1.02	10.00 ± 0.67	4.40 ± 0.92	86.25 ***
	10	8.80 ± 0.48	5.60 ± 0.24	4.00 ± 0.24	88 ***
	20	8.00 ± 0.63	4.40 ± 0.24	3.00 ± 0.19	91 ***

Significant at highly significant at *** *p* < 0.01.

**Table 4:** In vivo antipyretic activity of *H. rariflorum* stocks.

Treatment	Dose (mg/kg) Body Weight	Body Temperature (°C) (Mean ± SEM)				
		Normal	After Yeast Injection (0 h)	After 1 h	After 2 h	After 3 h
**Normal saline (10 mL/kg)**		37.00 ± 0.19	38.29 ± 0.08	38.17 ± 0.16	38.00 ± 0.04	38.00 ± 0.03
Paracetamol	20	37.00 ± 0.05	38.04 ± 0.02	37.22 ± 0.01	37.00 ± 0.21	36.04 ± 0.01
ZnO-NPs	5	37.08 ±0.03	37.49 ± 0.22	37.00 ± 0.16	37.50 ± 0.16	37.24 ± 0.11 ***
	10	37.11 ± 0.03	37.48 ± 0.16	37.00 ± 0.10	36.22 ± 0.12	36.10 ± 0.223 ***
	20	37.10 ± 0.04	37.30 ± 0.08	36.14 ± 0.044	36.11 ± 0.02	36.03 ± 0.01 ***

Significant at highly significant at *** *p* < 0.01.

**Table 5:** In vivo sedative activity of *H. rariflorum* stocks.

Treatments	Dose (mg/kg) Body Weight	Number of Lines Crossed in 10 min (Mean ± SEM)	Movement Inhibition (%)
Normal saline (10 mL/kg)		127.00 ± 0.67	0
Diazepam	0.5	5.00 ± 0.31	96.0
ZnO-NPs	5	43.00 ± 1.93	66.0
	10	38.20 ± 1.77	70.0
	20	23.20 ± 0.58	82.0

## Data Availability

The data that support the findings of this study are available on request from the corresponding author.
